# Nano-Therapies for Glioblastoma Treatment

**DOI:** 10.3390/cancers12010242

**Published:** 2020-01-19

**Authors:** Edouard Alphandéry

**Affiliations:** 1 Institut de Minéralogie, de Physique des Matériaux et de Cosmochimie, IMPMC, Sorbonne Université, Muséum National d’Histoire Naturelle, UMR CNRS 7590, IRD Place Jussieu, 75005 Paris, France; edouardalphandery@hotmail.com; Tel.: +33-632-697-020; 2 Nanobacterie SARL, 36 boulevard Flandrin, 75116 Paris, France; 3 Institute of Anatomy, UZH University of Zurich, Institute of Anatomy, Winterthurerstr. 190, CH-8057 Zurich, Switzerland

**Keywords:** nanoparticle, nanomedicine, glioblastoma, GBM, oncology, nanotechnology, tumor targeting, enhanced permeability and retention (EPR), blood brain barrier

## Abstract

Traditional anti-cancer treatments are inefficient against glioblastoma, which remains one of the deadliest and most aggressive cancers. Nano-drugs could help to improve this situation by enabling: (i) an increase of anti-glioblastoma multiforme (GBM) activity of chemo/gene therapeutic drugs, notably by an improved diffusion of these drugs through the blood brain barrier (BBB), (ii) the sensibilization of radio-resistant GBM tumor cells to radiotherapy, (iii) the removal by surgery of infiltrating GBM tumor cells, (iv) the restoration of an apoptotic mechanism of GBM cellular death, (v) the destruction of angiogenic blood vessels, (vi) the stimulation of anti-tumor immune cells, e.g., T cells, NK cells, and the neutralization of pro-tumoral immune cells, e.g., T_reg_ cells, (vii) the local production of heat or radical oxygen species (ROS), and (viii) the controlled release/activation of anti-GBM drugs following the application of a stimulus. This review covers these different aspects.

## 1. Introduction

Glioblastoma is one of the most aggressive and difficult to treat cancers. It is characterized by a life expectancy following diagnosis of only 12–18 months [1]. Standard treatments are ineffective for a number of reasons, such as the incapacity of surgery to remove all glioblastoma multiforme (GBM) tumor cells, notably the infiltrative ones, the difficulty for chemo-therapeutic drugs to reach the tumor, due to the blood brain barrier (BBB) that prevents them from diffusing towards the tumor, and the limitations of radiotherapy, which cannot easily eradicate radio-resistant GBM cells, notably stem cell ones. To these difficulties, the very particular location of GBM should be added, which makes it difficult to eradicate GBM cells while avoiding damaging healthy brain cells.

To overcome these hurdles, the use of nanoparticulate anti-GBM drugs has been suggested. The interest of nano-formulated drugs for cancer treatment has been reviewed elsewhere [2,3,4]. These drugs first improve the targeting of tumor cells by: (i) promoting the drug diffusion through the blood brain barrier, (ii) specific tumor targeting mechanisms relying on an enhanced permeability and retention (EPR) effect, with molecules attached to nano-drugs that bind tumor cell receptors, and diffusion of these nano-drugs towards the tumor by application of a magnetic field gradient, and (iii) a homogeneous distribution of anti-GBM drugs within the tumor, notably using convection enhanced delivery. Nano-formulations also increase the efficacy of anti-GBM drugs through multiple mechanisms of antitumor activities, such as: (i) improved efficacy of chemo/gene therapeutic drugs, in particular by promoting cellular internalization of these drugs, (ii) a radio-sensitizing effect, which increases the efficacy of radio-therapy against GBM tumor, (iii) immune mechanisms relying on activation of anti-tumor immune cells, e.g., T cells, NK cells, and/or deactivation of pro-tumor immune cells, e.g., T_reg_ cells, (iv) destruction of angiogenic blood vessels, (v) local production of heat or radical oxygen species (ROS), (vi) illumination of GBM tumor border to ease GBM removal by surgery, (vii) a Trojan horse method in which anti-tumor drugs enter GBM by escaping the monitoring/defense system of the tumor, and (viii) restoration of the GBM cell death apoptotic pathway. The purpose of this review is to describe these different anti-GBM nano-drugs and their mechanisms of action, and to highlight their advantages compared with non-nanoparticulate systems. This review is broader in scope than previous ones [4,5,6], which focus on nanoparticle (NP) BBB penetration and specific types of nanomaterials (NM). It describes more types of NM, in particular metallic ones, which can play an essential role in fighting against GBM disease.

## 2. Different Types of Nano-Systems for GBM Treatment

**Figure 1:**
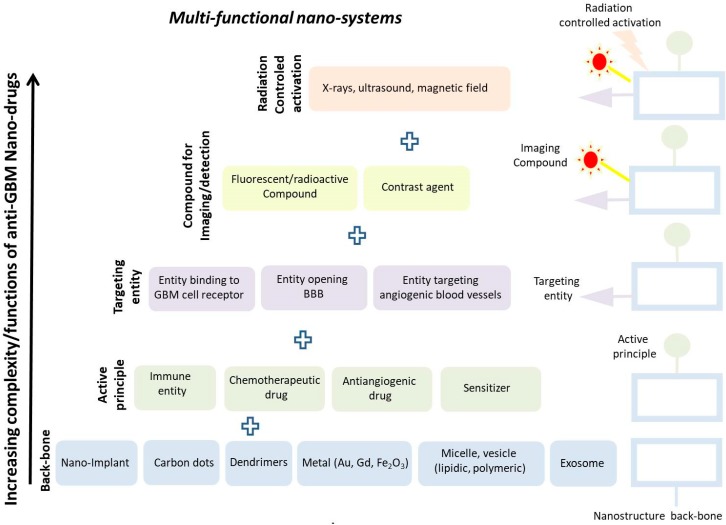
A schematic showing how a nanoparticulate system can be built up to include various functionalities to fight against GBM disease, such as: (i) a backbone, (ii) an active principle, (iii) a targeting moiety, and (iv) a compound used for imaging/detection.

Nano-systems are often used or presented as nanometric platforms with a backbone made of various elements such as vesicles (lipidic, micellar, polymeric or exosomes), linear polymers, metals (Au, Gd, graphene), carbon dots, nano-implants, dendrimers [7], inside or at the surface of which are inserted both active anti-GBM principles such as immune cells, chemotherapeutic/anti-angiogenic drugs or sensitizers, as well as moieties, which either target GBM cellular receptors/angiogenic blood vessels or open the BBB, and therefore help the active principles to reach GBM tumor cells [8]. These nano-systems can also be associated with fluorescent/radioactive compounds to enable their localization in the organism [9]. On top of that, it may in some cases be possible to activate these nano-systems on demand by deciding to apply (or not) an external source of energy such as X-rays, ultrasounds, or alternating magnetic field [10]. Figure 1 summarizes how such structures are built up, while Table 1 presents various nano-systems used for GBM treatment, with their backbone composition, functionalization/coating, mechanism of action, and efficacy demonstrated in vitro and/or in vivo. To the knowledge of the author, nano-systems are the only structures that combine so many different functionalities within a single drug unit, explaining the surge of interest that they have triggered. As an example, a nano-complex comprising a nanoparticulate backbone (PAMAM) associated with a targeting agent (RGD) and a chemotherapeutic drug (ATO), designated as RGDyC-mPEG-PAMAM/ATO, led to a decrease in GBM tumor volume, which was about two and four times larger than that reached with PAM associated with ATO (mPEG-PAMAM/ATO) and free ATO, respectively [11]. However, the above presentation does not take into consideration the following considerations. First, the nanoparticle backbone can have in itself the various functionalities described above without needing additional compounds. This can be the case when the backbone produces anti-tumor activity through the generation of an immune response [12] or ROS [13] (e.g., following its dissolution in the organism), or when it targets the tumor by being directly administered in the tumor or by passively diffusing towards the tumor via the EPR effect [14], or when it acts as contrast agent due to its composition/size/crystallinity, as is the case for iron oxide nanoparticles [15]. Second, the multiple functions of nano-systems are interdependent with each other (e.g., anti-GBM activity directly depends on the targeting efficacy of these drugs). Third, the binding/interacting properties of the compounds with the nanoparticle backbone are often not examined, and in many cases it is possible that the observed anti-GBM activity is due to the nanoparticle backbone and not to the compound, which has either detached from the backbone or has been destroyed in vivo. Therefore, although nano-system multi-functionality is an enormous advantage that will certainly help improve anti-GBM drug efficacy, it is difficult to determine with certainty its origin and to associate it with the various activities of the multiple components that it comprises. It may therefore be advisable to explore the multiple functions of the nanoparticle backbone and foresee its use as a single component before adding multiple compounds to it, whose functions are difficult to establish with certainty and that cannot easily be integrated in a nanoparticulate formulation using a good manufacturing practice (GMP) pharmaceutical fabrication process. Such reasoning is supported by the results obtained with simple nanoparticulate systems lacking therapeutic/targeting agents, which yielded strong anti-GBM activity, as shown for purified magnetosomes introduced in mouse GBM tumors and exposed to several alternating magnetic field (AMF) applications, leading to full tumor disappearance in treated GBM-bearing mice [16].

The activity of such nano-drugs can also in some cases be controlled by the application of an external source of energy (X-rays, ultrasound, magnetic field).

To be usable on humans, nanoparticles should be stable and release their active principle in a controlled fashion, two aspects that necessitate some optimization.

## 3. Nano-Drugs Improve GBM Drug Delivery

**Figure 2:**
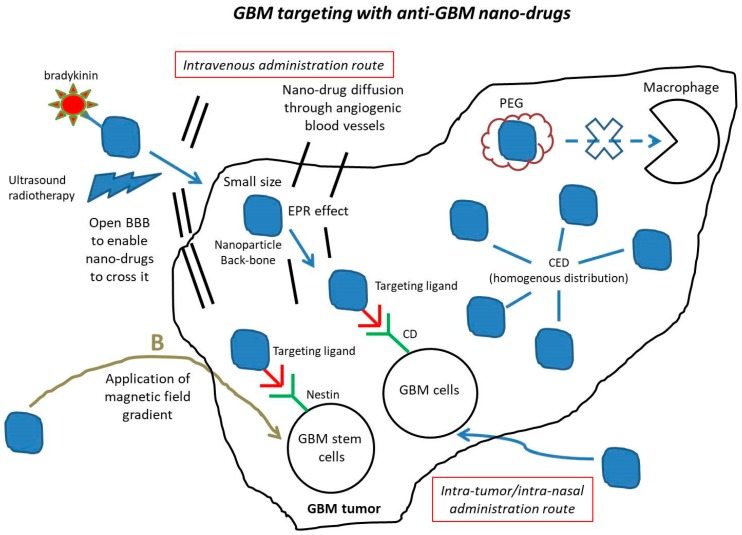
A schematic figure showing the various mechanisms by which anti-GBM nano-drugs can target the GBM tumor.

It is believed that anti-GBM drugs are not fully effective due to the existence of a series of barriers that prevent them from reaching these tumors. Such barriers include the BBB that blocks the entrance of drugs into GBM tumors, macrophages that can capture anti-GBM drugs, and anti-GBM drugs lacking a specific targeting mechanism to reach/bind GBM tumor cells, in particular GBM infiltrating/stem cells that play a major role in the development of this disease. In order to overcome these barriers, a series of strategies have been proposed, which are summarized below and in schematic Figure 2.

First, an intratumor administration route could be used to enable anti-GBM drugs to directly reach GBM tumors without having to cross the BBB. On the one hand, this can be achieved by injecting these drugs with a syringe or catheter at tumor location. It has been shown that nano-drugs administered in this way can remain in the tumor for a sufficiently long time to efficiently destroy the tumor (i.e., about one month) [16,65]. By contrast, intratumor administration of non-nano-formulated drugs is largely inefficient, resulting in drugs being either metabolized, eliminated [66], or diffusing towards several locations surrounding GBM tumors such as ventricles, subarachnoid space, cerebrospinal fluid or blood [67]. On the other hand, intratumor administration can be carried out with the help of convection enhanced delivery (CED), a technique following which anti-GBM drugs could diffuse at precisely controlled infusion rates towards GBM tumors under a hydrostatic pressure gradient using microcatheters implanted inside the tumor. While this method was reported to be largely inefficient to administer non-nano-formulated anti-GBM drugs in GBM tumors, it yielded for nano-formulated ones a homogenous distribution in the tumor and a half-life and diffusion volume larger than those of free drugs. These properties resulted in an enhanced anti-tumor efficacy, through sustained release of an active principle such as carboplatin from a nano-structure such as poly(lactic-co-glycolic acid) (PLGA) copolymer [68]. Furthermore, it has been shown that CED could result in antitumor efficacy at a much smaller dose of nanoparticles than both non-CED intratumor injection [69], and intravenous administration [70], an aspect that should receive full consideration given the high toxicity profile of chemotherapeutic drugs used for GBM treatments [71].

Second, another method to enable anti-GBM drugs reaching GBM tumors without being stuck by the BBB, consists of injecting these drugs by an intranasal route as carried out with Polyplex coated magnetite (Nano-co-Plex) loaded with BCNU or T7-targeted polyGIONs, enabling the active principles, miRNAs or BCNU, to efficiently reach GBM tumors [23,36].

Third, by applying MRI-guided focused ultrasounds [72] on the BBB, or by administering bradykinin [73,74], it has been possible to open or weaken this barrier and let anti-GBM drugs diffuse through it.

Fourth, passive targeting, also designated as EPR effect, can let the anti-GBM drugs diffuse through the holes of the blood vessels irrigating GBM tumors. To optimize the efficacy of this targeting, nano-drugs can either be coated with a substance such as polyethylene glycol (PEG) to prevent their capture by macrophages [75], or be of sufficiently small size (i.e., typically below 5 nm) to enable their diffusion through the holes of the angiogenic blood vessels [76].

Fifth, active targeting can be achieved by attaching substances to nanoparticles that specifically target a part of GBM tumor cells [6], such as an antigen (i.e., A2B5), differentiation clusters (i.e., CD15, CD33, CD44, or CD133), receptors of cytokines (i.e., interleukin13 receptor), and several proteins (i.e., Integrin-a6, α_5_β_3_, ανβ_3_ or L1CAM), which are expressed in GBM cells and can promote tumorigenesis. Examples of such substances include CTX, Pep-1, CBP4 and RGD, targeting CIC-13 chloride channel/matrix metalloproteinase (MMP-2), Interleukin13 receptor, CD33 and α_5_β_3_/ανβ_3_, respectively [48,71,77].

Sixth, magnetic targeting is an interesting concept, which relies on the application of a magnetic field directly on magnetic anti-GBM drugs, to trigger the diffusion of these drugs towards the tumor. Although this approach has shown some efficacy on small animals (e.g., magnetic anti-GBM drugs accumulated in rat glioblastoma following the application of a 0.5 T magnetic field [78]), its translation for human applications faces certain difficulties. On the one hand, equipment that generates a sufficiently strong and precise magnetic field gradient to trigger the diffusion of anti-GBM drugs towards tumors is currently lacking. On the other hand, the existence of many biological mechanisms, such as those of the immune system, blood flow, solid tissue resistance and cellular internalization, makes it difficult to model the diffusion of nano-drugs in vivo by only taking into consideration the properties of the applied magnetic field.

Seventh, one of the most important aspects to consider in the treatment of GBM is the targeting of GBM stem/infiltrating cells. Indeed, the failure of conventional therapies against this disease largely comes from their inability to target such cells. By functionalizing Gold nanorods with Nestin, which has been identified as a marker of these cells, it has been possible to selectively internalize gold nanorods in these cells [79,80].

Regarding the delivery of drugs with the help of nanoparticles, one of its most delicate aspects, which requiring further studies, concerns the preservation of the drug efficacy during its transport to the tumor.

## 4. Mechanisms of Action of Anti-GBM Nano-Drugs

**Figure 3:**
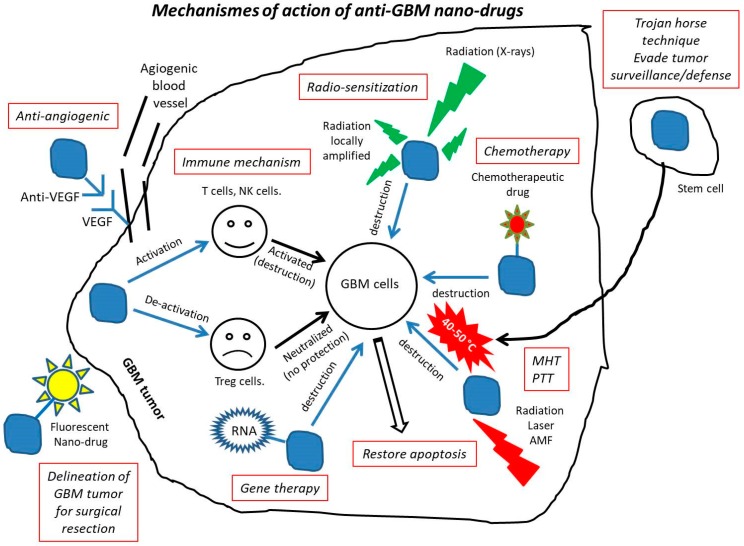
A schematic figure presenting the various mechanisms of action of anti-GBM drugs.

The different mechanisms of action of anti-GBM nano-drugs are summarized in Figure 3. Surgery, which is the first line of treatment recommended for removing operable GBM, can be improved by nano-therapy. Although this treatment enables removing a large part of the tumor, complete tumor surgical resection is almost impossible due to the infiltrative nature of GBM cells into surrounding normal brain tissues. Among the different methods suggested to improve the efficacy of surgery, the specific imaging of infiltrating GBM cells seems promising, since it enables visualizing them, which is a prerequisite for being able to remove them entirely. A series of different types of nano-systems have been developed to make this imaging method efficient. They work through: (i) the imaging of tumor-associated macrophages using near-infrared fluorescent silica coated iron oxide nanoparticles leading to the delineation of GBM tumor [33], (ii) a combined MR/fluorescent imaging method using targeting nanoparticles containing both MRI contrast (Gd-DTPA) and fluorescent dye (5-ALA) molecules [81], (iii) a nanostructure comprising indocyanine green (ICG) for NIR fluorescence and the trileucine peptide as fluorescence enhancer resulting in an intensive and long-lasting tumor fluorescence of human U87MG glioblastoma in mice and in an improved removal of these tumors [82].

Nano-formulated anti-GBM drugs can also improve the efficacy of chemotherapy by increasing the activity of various anti-tumor drugs, such as: (i) secondary metabolites of algae, which induced cytotoxicity towards A-172 glioblastoma cells when loaded in nano–microparticles [83], (ii) arsenic trioxide, which enabled treating temozolomide (TMZ)-resistant GBM cells following their encapsulation in liposomes in the presence of Mn [84], (iii) carboplatin, which led to higher tumor cytotoxicity, reduced neuronal toxicity and prolonged tissue half-life on rat and porcine GBM model when they were associated with PLGA copolymer [68], (iv) chlorotoxin, whose efficacy was increased compared with free drugs when they were conjugated with iron oxide nanoparticle, a behavior attributed to nanoparticles leading to a longer blood half-life, a better ability to cross the BBB and a faculty to be internalized in cells without losing its therapeutic activity [85], (v) cisplatin, which could move within the porous extracellular matrix between GBM cells when loaded with PEG-coated nanoparticles, yielding deeper brain penetration than non-pegylated cisplatin, and resulting in an increased survival of rats bearing GBM by more than two weeks compared with Cisplatin alone [66], (vi) curcumin, whose bioavailability and water solubility were increased when they were encapsulated in a dendrosome, suppressing U87MG cells growth without affecting healthy cells [86], (vii) doxorubicin, which led to remission among 20% of rats bearing GBM treated with DOX bound to polysorbate-coated nanoparticles [87], (viii) paclitaxel, which was more efficiently transported through the BBB and led to an improved anti-proliferative efficacy when it was associated with NPs and specific peptides Pep-1 designed to cross the BBB than when it was free [88], (ix) temozolomide (TMZ), which did not denature and could be specifically delivered to GBM cells with the help of the targeting peptide chlorotoxin (CTX), leading to enhanced cytotoxicity towards GBM cells compared with free TMZ [89].

Together with surgery and chemotherapy, radiotherapy is the third leg of GBM treatments. It has been shown that anti-GBM nano-formulations could enhance the effect of radiotherapy by: (i) increasing the downregulation of PD-L1 and EGFR using solid/lipid nanoparticles, resulting in a decrease of glioblastoma growth and prolonged mouse survival [41], (ii) enhancing the EPR effect, leading to better diffusion of nanoparticles to GBM tumors [90], (iii) sensitizing GBM cells to radiation by making GBM stem cells enter the radiation sensitive G2/M phase using the Sonic hedgehog ligand [91], by increasing DNA double-strand breaks using BSA–Au nanoparticles [92], or by exposing iodine nanoparticles to radiations [93], and (iv) enhancing the expression of the targets of CTX (i.e., MMP-2 and ClC-3), as well as BBB permeability and cellular internalization, leading to GBM tumor growth inhibition in vivo, using PLGA nanoparticles conjugated to chlorotoxin (CTX) [94]. The radio-sensitizing effect, which is often sought for when exposing nanoparticles to X-rays, is usually described as being optimal for nanoparticles of high atomic number Z, due to certain physical effects such as photoelectric ones, which are enhanced at high Z values. Such reasoning was used to justify the choice of nanoparticles composed of chemical elements with high Z values such as hafnium oxide. To be more complete, it should also probably take into account a whole set of other mechanisms that may occur during these nanoparticle/radiation interactions, such as other physical effects that could occur for low Z values (Compton effect), radiation amplification beyond nanoparticle surface, the formation of free radicals, nanoparticle distribution inside cells or in the extracellular matrix, nanoparticle interaction with biological material such as proteins, cell organelles, cell membranes and lipids, nanoparticle diffusion in liquids such as blood/cytoplasm or solids such as tissues, capture of nanoparticles by macrophages, stimulation of the immune system, and in vivo degradation, dissolution, disassembly or reassembly of nanoparticles.

Another commonly described mechanism of activation of anti-GBM nano-drugs relies on the release of such drugs under the application of various stimuli, such as:

1. Redox variations, which are due to a larger glutathione concentration inside than outside cells, where glutathione is responsible for cleavage of disulfide bonds [95], resulting in selective intracellular release of various chemotherapeutics from nanostructures (e.g., PTX from self-assembled nanoparticles [68] or DOX from polymeric micelles [54]).
2. pH, which is usually more acidic inside than outside the tumor, enabling the release of anti-GBM drugs from nanostructures when the linkers between them is acid-sensitive (e.g., DOX could be selectively released in tumors from polymeric micelles using poly(histidine) as linker [96]).
3. High intensity focused ultrasound, which can yield a higher accumulation of DOX in tumor tissue than in healthy tissue when applied on a nanostructure consisting of DOX attached to polymer [97].
4. Alternating magnetic field, which can trigger TMZ release from a lipid-based magnetic NM, and whose therapeutic activity against U87MG cells is enhanced by mild heating at 43 °C under these conditions of excitation [42].

Although these stimuli have been shown to increase anti-tumor efficacy in many studies, the mechanism by which the latter occurs is largely unknown, and drug release from the nanoparticle backbone, which is the most commonly brought forward explanation to account for such efficacy, has not been firmly demonstrated in vivo to the knowledge of the author.

When they are prepared in specific conditions (i.e., usually with a metal composition), nano-drugs can produce heat locally, essentially through the following two pathways:

1. Magnetic hyperthermia (MHT), in which magnetic nanoparticles are excited by an alternating magnetic field, producing localized nanoparticle heating, leading to anti-GBM efficacy in various GBM models (i.e., cellular, pre-clinical, and clinical ones) [98,99].
2. Photothermal therapy (PTT), where nanoparticles such as gold or iron oxide are exposed to near infra-red light (e.g., at 808 nm), absorbing this light and transforming it into localized heat through plasmonic effect, leading to the selective photothermal destruction of GBM cells at typical temperatures of 50–60 °C [35,100,101,102,103].

To foresee a widespread use of these techniques, MHT would first require installation of medically compatible induction coils in various hospitals, while PTT would need to overcome the drawbacks of low penetration depth and small exposed surface areas reached with the commonly used optical fibers to carry laser light.

In addition to heat, local perturbation can also be due to radical oxygen species (ROS) produced by anti-GBM nano-drugs. Most often, this effect is achieved using the photodynamic therapy (PDT) technique in which a photosensitizer such as porphyrin is exposed to laser light to generate ROS. The advantage of using the PDT technique in the presence of a nanoparticulate system comes from the control of ROS production that it enables (i.e., in theory ROS generation occurs at the nanoparticle location). As an example, mitochondrial-targeted photosensitizer-loaded albumin nanoparticles showed an enhanced cellular uptake and greater phototoxicity towards GBM cells than healthy cells *in vitro*, as well as a faculty to accumulate in GBM tumor and yield significant tumor suppression in a mouse GBM tumor model [104].

Another important type of anti-GBM treatment is gene therapy (RNA), which uses different types of RNA associated to nanoparticulate systems such as double-stranded RNA, siRNA, miR-101, resulting in an enhanced apoptosis of GBM cells as well as an inhibition of growth and migration of these cells through the targeting of specific genes (miR-34) or proteins (SOX9 or RAS) involved in the regulation/arrest of cellular pathways/cycles [61,64,105,106].

It has also been suggested to use the immune system to fight against GBM. Following this approach, nanotechnologies can yield certain improvements compared with non-nanoparticular treatments such as: (i) a better delivery in the tumor of immune entities such as checkpoint inhibitor antibodies to cytotoxic T-lymphocyte associated antigen 4 (a-CTLA-4) or programmed cell death-1 (a-PD-1), notably by enabling them to cross the BBB, resulting in an increase of T lymphocytes and NK cells, a decrease of regulatory T cells (T_regs_), and an increased survival of GBM-bearing mice [107], (ii) the delivery of different immune entities either simultaneously [108] or within the immune-suppressive tumor microenvironment (TME), leading to an increase of CD8+ T cells when sHDL nano-discs associated with CpG were delivered in GBM animal tumors [109], and (iii) the switching of tumor-associated macrophages (TAM) from a pro-GBM to an anti-GBM activity, a behavior that was observed when TAM were loaded with nano-diamonds bearing doxorubicin [54].

Furthermore, nanotechnologies can help fighting against angiogenesis, a phenomenon in which blood vessels that irrigate GBM tumors are more numerous and abnormal compared with normal blood vessels, supporting the progression, infiltration and migration of GBM cells. This can be achieved by nano-drugs promoting the growth of new non-angiogenetic vessels [110], or neutralizing certain angiogenetic growth factors including members of the vascular endothelium growth factor (VEGF) family [111], Angiogenin [112], or Tetrac [113], in particular, to improve the results obtained with non-nanoparticulate formation containing certain anti-angiogenic compounds such as anti-VEGF that failed to show a therapeutic benefit in clinical trials (phase III) [114]. More specifically, it has been demonstrated that complexes formed by Angiogenin (ANG) and gold nanoparticles (AuNPs) resulted in anti-angiogenic behavior towards GBM tumor cells [112]. Nano-formulated Tetrac led to devascularization of GBM tumor vessels (i.e., a 95% loss of tumoral vascularity in mice bearing GBM xenografts) [103], while graphite/graphene nanoparticles yielded a decreased concentration of intracellular ROS and RNS, hence affecting a series of mechanisms involved in the promotion of angiogenesis [115].

Tumor microenvironment (TME) is known to prevent most anti-GBM therapies from being fully efficient, through very complex and yet partly unknown properties. Indeed, TME is not only characterized by the presence of angiogenesis, but also by hypoxia, mild acidity, specific redox reactions, high interstitial pressure and dense stromal structure. When a nanoparticle based anti-GBM therapeutic approach is considered, such properties should be fully taken into consideration, in particular for reaching a homogenous NP distribution, which can be strongly affected by TME properties [96,116,117]. Methods to control nanoparticle distribution/activity within TME should be developed to achieve efficient anti-GBM treatment with nano-drugs.

Due to their self-renewability, high tumorigenicity, infiltrative behavior and radio/chemo resistance, GBM stem cells are thought to be responsible for the recurrence/persistence of GBM disease. Several nanoparticle-based approaches have hence been proposed to eradicate these cells. They are based on: (i) an enhanced response to irradiation due to a faculty of internalization of certain nanoparticulate systems such as chitosan-capped gold nanoparticles, enabling concentrating anti-GBM drugs inside cells [118], or due to the inhibition of CSC specific pathways/receptors [119], (ii) reducing the tumor-propagating human cancer stem cells through intracellular delivery of anti-GBM drugs such as miRNA, specifically inside GBM CSC, using nanoparticle-drug complex such as bioreducible poly(β-amino ester) nanoparticles associated with miRNA [63], (iii) inhibiting autophagy, resulting in a decrease of stemness-associating genes (SOX2, POU5F1 and NANOG) using nanoparticles linked to PTX and chloroquine (CQ) [120], or (iv) preventing hemolysis induced by certain anti-GBM drugs and hence enhancing cytotoxicity towards GBM stem cells through the use of a nanoplatform consisting of chitosan nanoparticles associated with anti-hemolytic 1,3β-Glucan and paclitaxel as chemotherapeutic drug [121].

The Trojan horse technique consists of inserting foreign entities in tumors to destroy them while minimizing identification, detection or destruction of these entities by the tumor surveillance/defense system, where such system is most often described as being immunologic [107]. It can consist of immune-conjugates [107] or stem cells containing anti-GBM drugs [122], with the aim of improving the specific release of anti-GBM drugs in tumors.

A final consideration concerns apoptosis, a mechanism of cellular death that could be weakened among GBM tumor cells, possibly making these cells resistant to standard treatments [123]. With the help of nanoparticulate systems, it has been shown that apoptosis could be restored, for example by heating magnetic nanoparticles in the presence of GBM cancer cells under the application of an alternating magnetic field at mild temperatures of 40–50 °C [1,16,42].

Regarding the mechanisms of action, it is of common practice to classify them in different categories and to identify a dominant one, which is brought forward to explain the origin of NP anti-tumor activity. However, the reality is probably different, being a superposition of different mechanisms coexisting together.

To conclude, I have highlighted various mechanisms of nano-drugs against GBM. While the diversity of these mechanisms is a considerable advantage to promote the emergence of an efficient anti-GBM treatment, it also requires identifying the most efficient one. Indeed, such identification could enable the use of a single drug unit triggering an optimal anti-GBM mechanism, which would be easier to develop than a drug comprising multiple compounds designed to neutralize various GBM tumorigenic functions simultaneously. However, this necessitates standardized pre-clinical tests to be able to compare the efficacy of the different anti-GBM drugs under development worldwide.

Furthermore, I have explained that nano-therapeutics present a number of appealing features that could help fighting GBM disease such as: (i) the faculty of these nano-drugs to diffuse through the BBB, targeting GBM tumors by active or magnetic targeting and hence efficiently reaching GBM tumors, or (ii) an enhanced anti-tumor activity through various local mechanisms (e.g., stimulation of the immune system, the generation of ROS, or the excitation of these drugs by an external source of energy). However, some of the barriers to reach an efficient GBM treatment do not depend on the type of tested therapy. Indeed, they are due to: (i) a lack of preclinical models that are close enough to human GBM, (ii) the difficulty in carrying out clinical trials on a sufficiently large number of patients to reach statistical significance of the clinical data, (iii) the design of clinical trials that plan to treat GBM patients at a too advanced stage of the disease, and (iv) the too late detection of GBM disease. To develop an efficient treatment against GBM requires overcoming all these various hurdles. This is the reason why this task is so difficult.

## Figures and Tables

**Table 1:** A summary of in vitro/in vivo anti-glioblastoma multiforme (GBM) activities reported in the literature for various nano-drugs with different compositions, abilities to bypass (or not) the blood brain barrier (BBB), and mechanisms of actions. NA: not available. Iv: intravenous. It: intratumoral. In: intranasal; IONP: Iron oxide nanoparticle

Nanoparticle Backbone Composition	Size (nm)	Functionalization Coating	Bypass BBB	In Vitro Efficacy	In Vivo Efficacy	Mech. of Action	Admin.	Ref.
Mesoporous silica	220	Curcumin + chrysin	NA	NA	NA	pH dependant release of curcumin/chrysin + cellular internalization	In	[17]
Mesoporous Silica	100–200	PDA+Asn-Gly-Arg (targ: CD13)	Yes	Cytotoxicity demonstrated on C6 cells	Increased survival in glioma bearing rates treated with NP	Increase accumulation in tumor tissue	Iv	[18]
Carbon nanotubes (CNT)	100–200	PEG	NA	Demonstrated on several cell lines (U87, U373, D54 NHA)	NA	Heat produced by CNT exposed to lIR aser	It	[19]
Carbon dots	6–8	NA	NA	Cytotoxicity on U87 cells	Increased survival of mice bearing U87 GBM tumors treated by CD exposed to IR laser	Heat produced by CD exposed to lIR aser	Iv	[20]
Carbon dots	2–4	transferrin (targeted ligand) + Epirubicin, temozolomide (anti-cancer drugs)	NA	Cytoxicity demonstrated on SJGBM2, CHLA266, CHLA200 and U87 GBM cells	NA	Target specifically GBM cells Enhances the effect of chemotherapy	NA	[21]
Iron oxide nanoparticle (IONP)	NA	PEI	NA	More cytotoxicity using MHT than exogenous heating	NA	Heat produced by IONP exposed to AMF	NA	[22]
IONP	30–50	Polyplex + BCNU	Yes	Cytotoxicity towards GBM cells	NA	Release of BCNU + internalization (intranasal admin)	NA	[23]
Fe_3_O_4_/Gd_2_O_3_	7	Cisplatin + lactoferrin + RGD	Yes	Cytotoxicity towards GBM cells	Increased survival of U87-Luc bearing mice treated with NP	Internalization in cancer cells and release of Fe^2+^, Fe^3+^ (Fenton reaction favored by cisplatin)	Iv	[24]
IONP	55	Chitosan	NA	Cytotoxoicity towards GBM cells (C6 and U87)	NA	Accumulation of NP in tumor following iv adminiistration	Iv	[25]
Magnetosome (IONP)	40	PEI, chitosan, neridronate	NA	Cytotoxicity demonstrated on GL261 and RG2 cells	NA	Heat produced by magnetosomes exposed to AMF	NA	[26]
Magnetosome (IONP)	40	Poly-L-lysine, citric acid, oleic acid, CMD	NA	Cytotoxicity demonstrated on GL261 and RG2 cells	NA	Heat produced by magnetosomes exposed to AMF	NA	[27]
Nanoparticle backbone composition	Size (nm)	Functionalization Coating	Bypass BBB	In vitro efficacy	In vivo efficacy	Mech. of action	Admin.	Ref.
Au NP	40	Fe_3_O_4_ + DNA	NA	Cytoxicity demonstrated on C6 cells	Decreased tumor growth in mice bearing C6 tumor treated by NP exposed to laser	Heat produced by NP exposed to laser + gene therapy	Iv	[28]
Au NP	20–35	Ala-Ala-Asn- Cys-Lys or 2-cyano-6-aminobenzothiazole modified AuNPs + DOX	NA	Cytotoxicity on C6 cells	Increased survivavl for mice treated with NP	Nanoparticle aggregation that blocks exocytosis and nanoparticle backflow in blood stream	Iv	[29]
Au NP AuNRs@SiO2	75	RVG29; PEG	yes	Cytotoxicity towards N2a cells	Tumor growth delay in mice bearing Na2 tumors	Photothermal therapy (NP mimicking virus) Bypass BBB through interaction between RVG29 and AchR	It	[30]
Au	35	RGD	NA	NA	Enhanced accumulation in brain due to RGD	NA	Iv	[31]
Au	5	DNA	NA	Cytotoxicity towards CSC-like U251MG-P1 cells and GBM U251MG cells in the presence of radiation	NA	Radiosensitize GBM stem cells (enhancement of abnormal nuclei)	NA	[32]
Au	37	Silica coated	yes	In vitro uptake of NP in U87-MG	In vivo delineation of glioblastoma	Endocytosis by tumor associated macrophages. Enables delineation of GBM tumor margin by fluorescence	Iv	[33]
Au	30	Irridium inserted inside NP	NA	Cytotoxicity towards U87 Luc cells at very low NP concentration (< 0.5 µM) due to combined PTT/PDT	NA	Combination of cell imaging/ PTT/ PDT	NA	[34]
Au	130	Albumin	NA	NA	Decrease in tumor growth in mice bearing N2a tumor	Combination of: - albumin for biocompatibility - Gold for photothermy (passive targeting)	Iv	[35]
Au + IONP	30	Chitosan+miRNA + TMZ+PEG-T7 peptide	Yes (in admin)	NA	NP treatment leads to: (i) accumulation of miRNAs in GBM tumor; (ii) increased survival of mice bearing GM tumor	Increased activity of miRNA + TMZ	In	[36]
Gd (AGuIX)	3	polysiloxane	NA	NA	Improved survivakl of rats bearing 9L GBM treated with AGUIX + RT	Improves delineation of GBM tumor;	iv	[37]
Nanoparticle backbone composition	Size (nm)	Functionalization Coating	Bypass BBB	In vitro efficacy	In vivo efficacy	Mech. of action	Admin.	Ref.
Liposome (nano)	<50	Docetaxel	NA	Increased cytotoxicity on C6 glioma cells compared with free drug	NA	Increased DTX accumulation in brain compared with free drug	NA	[38]
Liposome (nano)	100–135	DOPA + DNA	NA	Cellular uptake in GBM cells via receptor LAT1	Increaed survival of mice treated with liposomes	Chemotherapy + immunotherapy	Iv	[39]
Liposome (thermo-responsive)	< 270	PCTX	Yes	Cytoxicity towards GBM cells increases between 37 and 39°C	NA	Release of drugs with increasing temperature	NA	[40]
Liposome	50	cyclic peptide iRGD + siRNA-EGFR + siRNA-PD-L1	Yes	Cytoxicity towards U87 and GL-261 cells	Increased survival of mice bearing GL-261 glioblastoma tumors.	Increased targeting (radiotherapy + RGD) + chemotherapy + immunotherapy	Iv	[41]
Liposome + IONP	50–100	Temozolomide	Yes	Cytotoxicity towards U87-Luc cella	NA	Heat under AMF application + drug release	NA	[42]
Liposome	100–150	RGD + TMZ + Vincristine	NA	Cytotoxicity towards U87 and T98G GBM cells	Tumor growth deay in mice tbearing U87 GBM tumors reated with NP	Specific targerting of GBM cells + drug release	Iv	[43]
Liposome	100–150	Ursolic acid + EGCG + MAN	Yes	Cytotoxicity towards C6 GBM cells	Inhibition of GBM C6 tumor growth	EGCG induce apoptosis of GBM cells. MAN for targeting. UA, anti-cancer drug.	Iv	[44]
Liposome	121	TMZ	NA	Cytotoxicity towards CNS-1 GBM cells	Increase survival of GBM bearing rats (lipsomal formulation more efficient than free TMZ)	Increases the anti-tumor efficacy of TMZ	it	[45]
Nanoparticle backbone composition	Size (nm)	Functionalization Coating	Bypass BBB	In vitro efficacy	In vivo efficacy	Mech. of action	Admin.	Ref.
Aptamer (ssDNA)	NA	DOX	NA	Aptamer causes more inhibition on targeted cells A-172 than non-targeted cells MCF-7.	NA	Selective targeting of GBM cells	NA	[46]
Dendrimer (3G3)	NA	Curcumin	NA	NP internalized inside tumor cells selectively within nuclei.	NA	Selective cytotoxicity towards GBM cells	Iv	[47]
Dendrimer	NA	Arg-Gly-Asp (RGDyC) + αvβ3 integrin targeting ligand + PEG + ATO	yes	More cytotoxic than free ATO on C6 cell lines	RGDyC-mPEG-PAMAM could enhance the antitumor of ATO to glioma, it provides a desirable strategy for targeted therapy of glioma.	selective release of ATO at acidic pH	Iv	[11]
Polymer (PLGA)	NA	Chlorotoxin + Morusin	NA	Cytotoxicity towards GI-1 and U87 cell lines.	NA	Specifically target chloride channels (CIC-3) and matrix metalloproteinase (MMP-2), present in GBM cells/environment. Cytoxicity through ROS production	NA	[48]
Polymer (PLGA)	100	Nano-graphene + 5-iodo-2-deoxyuridine (IUdR)	NA	NP cause damage towards U87MG cell line in the presence of Xray (6 MV) and NIR laser.	NA	Reduced the plating efficiency of the cells Specific targeting of GBM cells	NA	[49]
Polymer (albumin)	150	paclitaxel and fenretinide	yes	Cytotoxicity towards U87 cells	Tumor growth delay and increased survival in mice bearing U87 GBM tumors.	Croosing of BBB Release of PTX	Iv	[50]
Polymer (poly(amine-co-ester) terpolymer)	NA	BBB modulator Lexiscan, NECA, minoxidil)	yes	NA	Increased survival of mice bearing intracranial GL-261 GBM	Accumulation in brain tumor and trigger gene therapy/chemotherapy	Iv	[51]
Polymer (PLGA-PLA-PCL) (nano-implant)	NA	TMZ	NA	NA	Increased survival of rats bearing GBM	Release of TMZ under laser excitation	NA	[52]
Polymer (Methylene Blue Oleate Salt-Loaded Polymeric NP)	170	Methylene blue	Yes	NPs inhibit U87 and T98G cells	NPs bypass BBB more efficientlt than free drugs.	Drug release in GBM tumor	iv	[53]
Nanoparticle composition	Size (nm)	Functionalization Coating	Bypass BBB	In vitro efficacy	In vivo efficacy	Mech. of action	Admin.	Ref.
Micelle (PEtOz-SS-PCL)	100–150	DOX	yes	Cytotoxicity towards C6 cells	Efficacy shown on orthotopic C6-Luci cells-bearing mice	Overcomes BBB and enhances DOX effect (release of DOX inside cells)	Iv	[54]
Micelle	80	BCNU + T7 peptide	yes	Cytotoxicity towards U87 GBM cells	Increased survival of mice bearing U87 GBM tumors	Accumulation of NP in tumor Increase in drug efficacy BCNU	Iv	[55]
Micelle (polymeric)	25	RI-VAP + D-VAP (targeting); paclitaxel (drug)	na	Cytotoxicity towards U87 and HUVEC cells	Delays tumor growth of mice bearing U87 GBM tumors	Targets tumors + release drug	Iv	[56]
Micelle	NA	panobinostat	Yes	Cytotoxicity towards F98, MO59K and U87-MG GBM cells	Increased survival of rats bearing GBM tumors	Inhibition of pan-histone deacetylase enhanced by NP	it	[57]
Micelle (FA-PEG-PCL)	20	luteolin	yes	Enhanced growth inhibition and more apoptosis of GL261 cells with NP	Increases survival of mice bearing GL261 GBM tumor	Increases the effect of luteolin	NA	[58]
Micelle (MPEG-PCL)	50	luteolin	NA	Luteolin/MPEG-PCL micelles had stronger cytotoxicity and induced a higher percentage of apoptosis in C6 and U87 cells than free luteolin Apotosis induced through mitochondrial pathway	Tumor growth delay in mice bearing C6 GBM tumors.	release of luteolin Luteolin/MPEG-PCL micelles induced more glioma cell apoptosis than free luteolin and inhibited neovascularization in tumor tissues	Iv	[59]
Micelle (Au+IONP)		PEG	NA	Increase in cell DNA damage when GBM cells are incubated with NP and irradiated at 4 Gy	Possibility to image tumor border by MRI (T2 contrast)	Radiosensitization: increase in DNA breaks when NP irradiated.	iv	[60]
RNA (+ MNP)	10	PEI	NA	No cytotoxicity	NA	Immune response against the tumor	NA	[61]
RNA (+ lipoprotein)	20–40	none	yes	Cytotoxicity towards C6 cells	Improved survival on mice bearing patient derived GICs glioblastoma	RNA-interfering efficiency, increases glioblastoma cell apoptosis	Iv	[62]
miRNAs (+ polymer NP)	100	none	NA	Cytotoxicity towards GBM cells	Treated mice bearing GBM have long term survival	Increase the efficacy of radiotherapy	It	[63]
miRNA (+ nanogel)	200	polyglycerol	NA	Cytotoxicity towards U87-Luc cells	Tumor growth delay observed in mice bearing xenograft U87 GBM tumors.	Gene targeting responsible for tumor cell suppression	It	[64]
RNAi (+ liposome)	NA	NA	NA	Reduction of GBM tumor sphere formation by NP	Prolonges survival of mice bearing GBM tumors	Target brain tumor-initiating cells	it	[62]

## Abbreviations

AMF	Alternating magnetic field
ATO	Arsenic trioxide
Au	Gold
BBB	Blood brain barrier
BCNU	Carmustine
CED	Convection enhanced delivery
CQ	Chloroquine
CTX	Chlorotoxin
CSC	Cancer stem cells
DOX	Doxorubicin
EPR	Enhanced permeability and retention effect
Gd	Gadolinium
GBM	Glioblastoma multiforme
GMP	Good manufacturing practice
MHT	Magnetic hyperthermia, the application of an alternating magnetic field on magnetic nanoparticles
NK	Natural killer
PTX	Paclitaxel
PAM	Polyacrylamide
PAMAM	Poly(amidoamine)
PDT	Photodynamic therapy, the application of a laser light on a photosensitizer to produce ROS
PLGA	Poly(lactic-co-glycolic acid)
PEG	Polyethylene glycol
PTT	Photothermal therapy, the application of laser light on nanoparticles, producing heat through plasmonic effects
RGD	Arginylglycylaspartic acid peptide
RNS	Radical nitrogen species
ROS	Radical oxygen species
T cells	T lymphocytes
TMZ	Temozolomide
TME	Tumor microenvironment
VEGF	Vascular endothelium growth factor

## References

[1] Alphandéry E. (2018). Glioblastoma Treatments: An Account of Recent Industrial Developments. Front. Pharmacol..

[2] Jahanban-Esfahlan A., Seidi K., Jaymand M., Schmidt T.L., Majdi H., Javaheri T., Jahanban-Esfahlang M., Zarei P. (2019). Dynamic DNA nanostructures in biomedicine: Beauty, utility and limits. J. Control. Release.

[3] Patra J.K., Das G., Fraceto L.F., Campos E.V.R., del Pilar Rodriguez-Torres M., Acosta-Torres L.S., Diaz-Torres L.A., Grillo R., Swamy M.K., Sharma S. (2018). Nano based drug delivery systems: Recent developments and future prospects. J. Nanobiotechnol..

[4] Jahanban-Esfahlan R., Seidi K., Jahanban-Esfahlan A., Jaymand M., Alizadeh E., Majdi H., Najjar R., Javaheri T., Zare P. (2019). Static DNA Nanostructures for Cancer Theranostics: Recent Progress in Design and Applications. Nanotechnol. Sci. Appl..

[5] Fakhoury M. (2016). Drug delivery approaches for the treatment of glioblastoma multiforme. Artif. Cells Nanomed. Biotechnol..

[6] Glaser T., Han I., Wu L., Zeng X. (2017). Targeted Nanotechnology in Glioblastoma Multiforme. Front. Pharmacol..

[7] Prasad M., Lambe U.P., Brar B., Shah I., Manimegalai J., Ranjan K., Rao R., Kumar S., Mahant S., Khurana S.K. (2018). Nanotherapeutics: An insight into healthcare and multi-dimensional applications in medical sector of the modern world. Biomed. Pharmacother..

[8] Wadajkar A.S., Dancy J.G., Hersha D.S., Anastasiadis P., Tran N.L., Woodworth G.F., Winkles J.A., Kim A.J. (2017). Tumor-targeted Nanotherapeutics: Overcoming Treatment Barriers for Glioblastoma. Wiley Interdiscip. Rev. Nanomed. Nanobiotechnol..

[9] Gonawala1 S., Ali M.M. (2017). Application of Dendrimer-based Nanoparticles in Glioma Imaging. J. Nanomed Nanotechnol..

[10] Sneider A., VanDyke D., Paliwal S., Rai P. (2017). Remotely Triggered Nano-Theranostics for Cancer Applications. Nanotheranostics.

[11] Lu Y., Han S., Zheng H., Ma R., Ping Y., Zou J., Tang H., Zhang Y., Xu X., Li F. (2018). A novel RGDyC/PEG co-modified PAMAM dendrimer-loaded arsenic trioxide of glioma targeting delivery system. Int. J. Nanomed..

[12] Yong S.B., Chung J.Y., Song Y., Kim J., Ra S., Kim Y.H. (2019). Non-viral nano-immunotherapeutics targeting tumor microenvironmental immune cells. Biomaterials.

[13] Yang B., Chen Y., Shi J. (2019). Reactive Oxygen Species (ROS)-Based Nanomedicine. Chem. Rev..

[14] Nel A., Ruoslahti E. (2017). New Insights into “Permeability” as in the Enhanced Permeability and Retention Effect of Cancer Nanotherapeutics. ACS Nano.

[15] Li L., Jiang W., Luo K., Song H., Lan F., Wu Y., Gu Z. (2013). Superparamagnetic Iron Oxide Nanoparticles as MRI contrast agents for Non-invasive Stem Cell Labeling and Tracking. Theranostics.

[16] Alphandéry E., Idbaih A., Adam C., Delattre J.-Y., Schmitt C., Guyot F., Chebbi I. (2019). Development of non-pyrogenic magnetosome minerals coated with poly-l-lysine leading to full disappearance of intracranial U87-Luc tumors in 100% of treated mice using magnetic hyperthermia. Biomaterials.

[17] Lungarea S., Hallam K., Badhana R.K.S. (2016). Phytochemical-loaded mesoporous silica nanoparticles for nose-to-brain olfactory drug delivery. Int. J. Pharm..

[18] Hu J., Zhang X., Wen Z., Tan Y., Huang N., Cheng S., Zheng H., Cheng Y. (2016). Asn-Gly-Arg-modified polydopamine-coated nanoparticles for dual-targeting therapy of brain glioma in rats. Oncotarget.

[19] Eldridge B.N., Bernish B.W., Fahrenholtz C.D., Singh R. (2016). Photothermal therapy of glioblastoma multiforme using multiwalled carbon nanotubes optimized for diffusion in extracellular space. ACS Biomater. Sci. Eng..

[20] Qian M., Du Y., Wang S., Li C., Jiang H., Shi W., Chen J., Wang Y., Wagner E., Huang R. (2018). Highly Crystalline Multicolor Carbon Nanodots for Dual-Modal Imaging-Guided Photothermal Therapy of Glioma. ACS Appl. Mater. Interfaces.

[21] Hettiarachchi S.D., Graham R.M., Mintz K.J., Zhou Y., Vanni S., Penga Z., Leblanc R.M. (2019). Triple conjugated carbon dots as a nano-drug delivery model for glioblastoma brain tumors. Nanoscale.

[22] Sanz B., Calatayud M.P., Torres T.E., Fanarrag M.L., Ibarra M.R., Goya G.F. (2017). Magnetic hyperthermia enhances cell toxicity with respect to exogenous heating. Biomaterials.

[23] Akiloa O.D., Choonara Y.E., Strydom A.M., du Toita L.C., Kumara P., Modic G., Pillaya V. (2016). An in vitro evaluation of a carmustine-loaded Nano-co-Plex for potential magnetic-targeted intranasal delivery to the brain. Int. J. Pharm..

[24] Shen Z., Liu T., Li Y., Lau J., Yang Z., Fan W., Zhou Z., Shi C., Ke C., Bregadze V.E. (2018). Fenton-Reaction-Acceleratable Magnetic Nanoparticles for Ferroptosis Therapy of Orthotopic Brain Tumors. ACS Nano.

[25] Shevtsov M., Nikolaev B., Marchenko Y., Yakovleva L., Skvortsov N., Mazur A., Tolstoy P., Ryzhov V., Multhoff G. (2018). Targeting experimental orthotopic glioblastoma with chitosan-based superparamagnetic iron oxide nanoparticles (CS -DX-SPIONs). Int. J. Nanomed..

[26] Hamdous Y., Chebbi I., Mandawala C., Le Fèvre R., Guyot F., Seksek O., Alphandéry E. (2017). Biocompatible coated magnetosome minerals with various organization and cellular interaction properties induce cytotoxicity towards RG-2 and GL-261 glioma cells in the presence of an alternating magnetic field. J. Nanobiotechnol..

[27] Mandawala C., Chebbi I., Durand-Dubief M., Le Fèvre R., Hamdous Y., Guyot F., Alphandéry E. (2017). Biocompatible and stable magnetosome minerals coated with poly-l-lysine, citric acid, oleic acid, and carboxy-methyl-dextran for application in the magnetic hyperthermia treatment of tumors. J. Mater. Chem. B.

[28] Hu Y., Zhou Y., Zhao N., Liu F., Xu F.-J. (2016). Multifunctional pDNA-Conjugated Polycationic Au Nanorod-Coated Fe 3 O 4 Hierarchical Nanocomposites for Trimodal Imaging and Combined Photothermal/Gene Therapy. Small.

[29] Ruan S., Hu C., Tang X., Cun X., Xiao W., Shi K., He Q., Gao H. (2016). Increased Gold Nanoparticle Retention in Brain Tumors by in Situ Enzyme-Induced Aggregation. ACS Nano.

[30] Lee C., Hwang H.S., Lee S., Kim B., Kim J.O., Oh K.T., Lee E.S., Choi H.-G., Youn Y.S. (2017). Rabies Virus-Inspired Silica-Coated Gold Nanorods as a Photothermal Therapeutic Platform for Treating Brain Tumors. Adv. Mater..

[31] Albertini B., Mathieu V., Iraci N., Van Woensel M., Schoubben A., Donnadio A., Greco S.M.L., Ricci M., Temperini A., Blasi P. (2019). Tumor Targeting by Peptide-Decorated Gold Nanoparticles. Mol. Pharm..

[32] Kunoh T., Shimura T., Kasai T., Matsumoto S., Mahmud H., Khayrani A.C., Seno M., Kunoh H., Takada J. (2019). Use of DNA-generated gold nanoparticles to radiosensitize and eradicate radioresistant glioma stem cells. Nanotechnology.

[33] Lee C., Kim G.R., Yoon J., Kim S.E., Yoo A.S., Piao X. (2018). In vivo delineation of glioblastoma by targeting tumor-associated macrophages with near-infrared fluorescent silica coated iron oxide nanoparticles in orthotopic xenografts for surgical guidance. Sci. Rep..

[34] Ricciardi L., Sancey L., Palermo G., Termine R., De Luca A., Szerb E.I., Aiello I. (2017). Plasmon-mediated cancer phototherapy: The combined effect of thermal and photodynamic processes. Nanoscale.

[35] Seo B., Lima K., Kim S.S., Oh K.T., Lee E.S., Choi H.-G., Shin B.S., Youn Y.S. (2019). Small gold nanorods-loaded hybrid albumin nanoparticles with high photothermal efficacy for tumor ablation. Colloids Surf. B Biointerfaces.

[36] Sukumar U.K., Bose RJ C., Malhotra M., Babikir H.A., Afjei R., Robinson E., Zeng Y., Chang E., Habte F., Sinclair R. (2019). Intranasal delivery of targeted polyfunctional gold–iron oxide nanoparticles loaded with therapeutic microRNAs for combined theranostic multimodality imaging and presensitization of glioblastoma to temozolomide. Biomaterials.

[37] Dufort S., Appelboom G., Verry C., Barbier E.L., Lux F., Bräuer-Krisch E., Sancey L., Chang S.D., Zhang M., Roux S. (2019). Ultrasmall theranostic gadolinium-based nanoparticles improve high-grade rat glioma survival. J. Clin. Neurosci..

[38] Shaw T.K., Dey D.M.G., Pal M.M., Paul P., Chakraborty S., Ali K.A., Mukherjee B., Bandyopadhyay A.K., Mandal M. (2017). Successful delivery of docetaxel to rat brain using experimentally developed nanoliposome: A treatment strategy for brain tumor. Drug Deliv..

[39] Bhunia S., Vangala V., Bhattacharya D., Ravuri H.G., Kuncha M., Chakravarty S., Sistla R., Chaudhuri A. (2017). Large Amino Acid Transporter 1 Selective Liposomes of L-DOPA Functionalized Amphiphile for Combating Glioblastoma. Mol. Pharm..

[40] Rehman M., Madni A., Shi D., Ihsan A., Tahir N., Chang K.R., Javed I., Webster T.J. (2017). Enhanced blood brain barrier permeability and glioblastoma cell targeting via thermoresponsive lipid nanoparticles. Nanoscale.

[41] Erel-Akbaba G., Carvalho L.A., Tian T., Zinter M., Akbaba H., Obeid P.J., Chiocca E.A., Weissleder R., Kantarci A.G., Tannous B.A. (2019). Radiation-Induced Targeted Nanoparticle-Based Gene Delivery for Brain Tumor Therapy. ACS Nano.

[42] Tapeinos C., Marino A., Battaglini M., Migliorin S., Brescia R., Scarpellini A., Fernández C.D.J., Prato M., Dragog F., Ciofani G. (2019). Stimuli-responsive lipid-based magnetic nanovectors increase apoptosis in glioblastoma cells through synergic intracellular hyperthermia and chemotherapy. Nanoscale.

[43] Zhang Y., Zhai M., Chen Z., Han X., Yu F., Li Z., Xie X., Han C., Yu L., Yang Y. (2017). Dual-modified liposome codelivery of doxorubicin and vincristine improve targeting and therapeutic efficacy of glioma. Drug Deliv..

[44] Ying X., Wang Y., Xu H., Li X., Yan H., Tang H., Wen C., Li Y. (2017). The construction of the multifunctional targeting ursolic acids liposomes and its apoptosis effects to C6 glioma stem cells. Oncotarget.

[45] Nordling-David M.M., Yaffe R., Guez D., Meirow H., Last D., Grad A., Salomon S., Sharabi S., Levi-Kalisman Y., Golomb G. (2017). Liposomal temozolomide drug delivery using convection enhanced delivery. J. Control. Release.

[46] Bayrac A.T., Akca O.E., Eyidogan F.I., Oktem H.A. (2018). Target-specific delivery of doxorubicin to human glioblastoma cell line via ssDNA aptamer. J. Biosci..

[47] Gamage N.H., Jing L., Worsham M.J., Ali M.M. (2016). Targeted Theranostic Approach for Glioma Using Dendrimer-Based Curcumin Nanoparticle. J. Nanomed Nanotechnol..

[48] Agarwal S., Muniyandi P., Maekawa T., Kumar D.S. (2018). Vesicular systems employing natural substances as promising drug candidates for MMP inhibition in glioblastoma: A nanotechnological approach. Int. J. Pharm..

[49] Kargar S., Khoei S., Khoee S., Shirvalilou S., Mahdavi S.R. (2018). Evaluation of the combined effect of NIR laser and ionizing radiation on cellular damages induced by IUdR-loaded PLGA-coated Nano-graphene oxide. Photodiagn. Photodyn. Ther..

[50] Lin T., Zhao P., Jiang Y., Tang Y., Jin H., Pan Z., He H., Yang V.C., Huang Y. (2016). Blood−Brain-Barrier-Penetrating Albumin Nanoparticles for Biomimetic Drug Delivery via Albumin-Binding Protein Pathways for Antiglioma Therapy. ACS Nano.

[51] Han L., Kong D.K., Zheng M.-Q., Murikinati S., Ma C., Yuan P., Li L., Tian D., Cai Q., Ye C. (2016). Increased Nanoparticle Delivery to Brain Tumors by Autocatalytic Priming for Improved Treatment and Imaging. ACS Nano.

[52] Ramachandran R., Junnuthula V.R., Gowd G.S., Ashokan A., Thomas J., Peethambaran R., Thomas A., Unni AK K., Panikar D., Nair S.V. (2017). Theranostic 3-Dimensional nano brain-implant for prolonged and localized treatment of recurrent glioma. Sci. Rep..

[53] Castañeda-Gill J.M., Ranjan A.P., Vishwanatha J.K. (2017). Development and Characterization of Methylene Blue Oleate Salt-Loaded Polymeric Nanoparticles and their Potential Application as a Treatment for Glioblastoma. J. Nanomed Nanotechnol..

[54] Li Y., Baiyang L., Leran B., Zhen W., Yandong X., Baixiang D., Dandan Z., Yufu Z., Jun L., Rutong Y. (2017). Reduction-responsive PEtOz-SS-PCL micelle with tailored size to overcome blood–brain barrier and enhance doxorubicin antiglioma effect. Drug Deliv..

[55] Bi Y., Liu L., Lu Y., Sun T., Shen C., Chen X., Chen Q., An S., He X., Ruan C. (2016). T7 Peptide-Functionalized PEG-PLGA Micelles Loaded with Carmustine for Targeting Therapy of Glioma. ACS Appl. Mater. Interfaces.

[56] Ran D., Mao J., Shen Q., Xie C., Zhan C., Wang R., Lu W. (2017). GRP78 enabled micelle-based glioma targeted drug delivery. J. Control. Release.

[57] Singleton W.G., Collins A.M., Bienemann A.S., Killick-Cole C.L., Haynes H.R., Asby D.J., Butts C.P., Wyatt M.J., Barua N.U., Gill S.S. (2017). Convection enhanced delivery of panobinostat (LBH589)-loaded pluronic nano-micelles prolongs survival in the F98 rat glioma model. Int. J. Nanomed..

[58] Wu C., Xu Q., Chen X., Liu J. (2019). Delivery luteolin with folacin-modified nanoparticle for glioma therapy. Int. J. Nanomed..

[59] Zheng S., Cheng Y., Teng Y., Liu X., Yu T., Wang Y., Liu J., Hu Y., Wu C., Wang X. (2017). Application of luteolin nanomicelles anti-glioma effect with improvement in vitro and in vivo. Oncotarget.

[60] Sun L., Chen Y., Zhou Y., Guo D., Fan Y., Guo F., Zheng Y., Chen W. (2017). Preparation of 5-fluorouracil-loaded chitosan nanoparticles and study of the sustained release in vitro and in vivo. Asian J. Pharm. Sci..

[61] Grabowska M., Grześkowiak B.F., Szutkowski K., Wawrzyniak D., Głodowicz P., Barciszewski J., Jurga S., Rolle K., Mrowczyński R. (2019). Nano-mediated delivery of double-stranded RNA for gene therapy of glioblastoma multiforme. PLoS ONE.

[62] Yu D., Khan O.F., Suvàc M.L., Dong B., Panek W.K., Xiao T., Wu M., Han Y., Ahmed A.U., Balyasnikova I.V. (2017). Multiplexed RNAi therapy against brain tumorinitiating cells via lipopolymeric nanoparticle infusion delays glioblastoma progression. Proc. Natl. Acad. Sci. USA.

[63] Lopez-Bertoni H., Kozielski K.L., Rui Y., La R., Vaughan H., Wilson D.R., Mihelson N., Eberhart C.G., Laterra J., Green J.J. (2018). Bioreducible Polymeric Nanoparticles Containing Multiplexed Cancer Stem Cell Regulating miRNAs Inhibit Glioblastoma Growth and Prolong Survival. Nano Lett..

[64] Shatsberg Z., Zhang X., Ofek P., Malhotr S., Krivitsky A., Scomparin A., Tiram G., Calderón M., Haag R., Satchi-Fainaro R. (2016). Functionalized nanogels carrying an anticancer microRNA for glioblastoma therapy. J. Control. Release.

[65] Alphandéry E., Idbaih A., Adam C., Delattre J.-Y., Schmitt C., Guyot F., Chebbi I. (2017). Chains of magnetosomes with controlled endotoxin release and partial tumor occupation induce full destruction of intracranial U87-Luc glioma in mice under the application of an alternating magnetic field. J. Control. Release.

[66] Zhang C., Nance E.A., Mastorakos P., Chisholma P., Berry S., Eberhart C., Tyler B., Brem H., Soo J.S., Hanes J. (2017). Convection enhanced delivery of cisplatin-loaded brain penetrating nanoparticles cures malignant glioma in rats. J. Control. Release.

[67] Young J.S., Bernal G., Polster S.P., Nunez L., Larsen G.F., Mansour N., Podell M., Yamini B. (2018). Convection Enhanced Delivery of Polymeric Nanoparticles Encapsulating Chemotherapy in Canines with Spontaneous Supratentorial Tumors. World Neurosurg..

[68] Arshad A., Yang B., Bienemann A.S., Barua N.U., Wyatt M.J., Woolley M., Johnson D.E., Edler K.J., Gill S.S. (2015). Convection-Enhanced Delivery of Carboplatin PLGA Nanoparticles for the Treatment of Glioblastoma. PLoS ONE.

[69] Finbloom J.A., Aanei I.L., Bernard J.M., Klass S.H., Elledge S.K., Han K., Ozawa T., Nicolaides T.P., Berger M.S., Francis M.B. (2018). Evaluation of Three Morphologically Distinct Virus-Like Particles as Nanocarriers for Convection-Enhanced Drug Delivery to Glioblastoma. Nanomaterials.

[70] Alphandéry E. (2019). Biodistribution and targeting properties of iron oxide nanoparticles for treatments of cancer and iron anemia disease. Nanotoxicology.

[71] Ganipineni P.L., Danhier F., Préat V. (2018). Drug delivery challenges and future of chemotherapeutic nanomedicine for glioblastoma treatment. J. Control. Release.

[72] Coluccia D., Figueiredo C.A., Wu M.Y., Riemenschneider A.N., Diaz Luck A., Smith C., Das S., Ackerley C., O’Reilly M., Hynynen K. (2018). Enhancing glioblastoma treatment using cisplatin-gold-nanoparticle conjugates and targeted delivery with magnetic resonance-guided focused ultrasound. Nanomed. Nanotechnol. Biol. Med..

[73] Azad T.D., Pan J., Connolly I.D., Remington A., Wilson C.M., Grant G.A. (2015). Therapeutic strategies to improve drug delivery across the blood-brain barrier. Neurosurg Focus.

[74] Parodi A., Rudzinska M., Deviatkin A.A., Soond S.M., Baldin A.V., Zamyatnin A.A. (2019). Established and Emerging Strategies for Drug Delivery Across the Blood-Brain Barrier in Brain Cancer. Pharmaceutics.

[75] Yu S.S., Lau C.M., Thomas S.N., Jerome W.G., Maron D.J., Dickerson J.H., Hubbell J.A., Giorgio T.D. (2012). Size- and charge-dependent non-specific uptake of PEGylated nanoparticles by macrophages. Int. J. Nanomed..

[76] Peng C., Gao X., Xu J., Du B., Ning X., Tang S., Bachoo R.M., Yu M., Ge W.-P., Zheng J. (2017). Targeting orthotopic gliomas with renal-clearable luminescent gold nanoparticles. Nano Res..

[77] Jiang Y., Wang X., Liu X., Lv W., Zhang H., Zhang M., Li X., Xin H., Xu Q. (2017). Enhanced Antiglioma Efficacy of Ultrahigh Loading Capacity Paclitaxel Prodrug Conjugate Self-Assembled Targeted Nanoparticles. ACS Appl. Mater. Interfaces.

[78] Shirvalilou S., Khoei S., Khoee S., Raoufi N.J., Karimi M.R., Shakeri-Zadeh A. (2018). Development of a magnetic nano-graphene oxide carrier for improved glioma-targeted drug delivery and imaging: In vitro and in vivo evaluations. Chem. Biol. Interact..

[79] Gonçalves D.P.N., Rodriguez R.D., Kurth T., Bray L.J., Binner M., Jungnickel C., Gür F.N., Poser S.W., Schmidt T.L., Zahn D.R.T. (2017). Enhanced targeting of invasive glioblastoma cells by peptidefunctionalized gold nanorods in hydrogel-based 3D culture. Acta Biomater..

[80] Gonçalves D.P.N., Park D.M., Schmidt T.L., Werner C. (2018). Modular peptide-functionalized gold nanorods for effective glioblastoma multicellular tumor spheroid targeting. Biomater. Sci..

[81] Ni D., Zhang J., Bu W., Xing H., Han F., Xiao Q., Yao Z., Chen F., He Q., Liu J. (2014). Dual-Targeting Upconversion Nanoprobes across the BloodBrain Barrier for Magnetic Resonance/Fluorescence Imaging of Intracranial Glioblastoma. ACS Nano.

[82] Patil R., Galstyan A., Sun T., Shatalova E.S., Butte P., Mamelak A.N., Carico C., Kittle D.C., Grodzinski Z.B., Chiechi A. (2019). Polymalic acid chlorotoxin nanoconjugate for near-infrared fluorescence guided resection of glioblastoma multiforme. Biomaterials.

[83] Karakaş C.Y., Şahin H.T., İnan B., Özçimen D., Erginer Y.Ö. (2019). In vitro cytotoxic activity of microalgal extracts loaded nano–micro particles produced via electrospraying and microemulsion methods. Biotechnol. Prog..

[84] Zhang L., Zhang Z., Mason R.P., Sarkaria J.N., Zhao D. (2015). Convertible MRI contrast: Sensing the delivery and release of antiglioma nano-drugs. Sci. Rep..

[85] Mu Q., Lin G., Patton V.K., Wang K., Press O.W., Zhang M. (2016). Gemcitabine and Chlorotoxin Conjugated Iron Oxide Nanoparticles for Glioblastoma Therapy. J. Mater. Chem. B.

[86] Mirgani M.T., Isacchi B., Sadeghizadeh M., Marra F., Bilia A.R., Mowla S.J., Najafi F., Babaei E. (2014). Dendrosomal curcumin nanoformulation downregulates pluripotency genes via miR-145 activation in U87MG glioblastoma cells. Int. J. Nanomed..

[87] Steiniger S.C.J., Kreuter J., Khalansky A.S., Skidan I.N., Bobruskin A.I., Smirnova Z.S., Severin S.E., Uhl R., Kock M., Geiger K.D. (2004). Chemotherapy of glioblastoma in rats using doxorubicin-loaded nanoparticles. Int. J. Cancer.

[88] Di Mauro P.P., Cascante A., Vilà P.B., Gómez-Vallejo V., Llop J., Borrós S. (2018). Peptide functionalized and high drug loaded novel nanoparticles as dualtargeting drug delivery system for modulated and controlled release of paclitaxel to brain glioma. Int. J. Pharm..

[89] Fang C., Wang K., Stephen Z.R., Mu Q., Kievit F.M., Chiu D.T., Press O.W., Zhang M. (2015). Temozolomide Nanoparticles for Targeted Glioblastoma Therapy. ACS Appl. Mater. Interfaces.

[90] Mi1 Y., Shao Z., Vang J., Kaidar-Person O., Wang A.Z. (2016). Application of nanotechnology to cancer radiotherapy. Cancer Nano.

[91] Morgenroth1 A., Vogg AT J., Ermert K., Zlatopolskiy B., Mottaghy F.M. (2014). Hedgehog signaling sensitizes Glioma stem cells to endogenous nano-irradiation. Oncotarget.

[92] Chen N., Yang W., Bao Y., Xu H., Qin S., Tu Y. (2015). BSA capped Au nanoparticle as an efficient sensitizer for glioblastoma tumor radiation therapy. RSC Adv..

[93] Hainfeld J.F., Ridwan S.M., Stanishevskiy1 Y., Panchal R., Slatkin1 D.N., Smilowitz H.M. (2019). Iodine nanoparticles enhance radiotherapy of intracerebral human glioma in mice and increase efficacy of chemotherapy. Sci. Rep..

[94] Tamborini M., Locatelli E., Rasile M., Monaco I., Rodighiero S., Corradini I., Franchini M.C., Passoni L., Matteoli M. (2016). A Combined Approach Employing Chlorotoxin-Nanovectors and Low Dose Radiation To Reach Infiltrating Tumor Niches in Glioblastoma. ACS Nano.

[95] Fernandes C., Suares D., Yergeri M. (2018). Tumor Microenvironment Targeted Nanotherapy. Front. Pharmacol..

[96] Uthaman S., Huh K.M., Park I.-K. (2018). Tumor microenvironment-responsive nanoparticles for cancer theragnostic applications. Biomater. Res..

[97] Yang F.-Y., Teng M.-C., Lu M., Liang H.-F., Lee Y.-R., Yen C.-C., Liang M.-L., Wong T.-T. (2012). Treating glioblastoma multiforme with selective high-dose liposomal doxorubicin chemotherapy induced by repeated focused ultrasound. Int. J. Nanomed..

[98] Chen L., Wu Y., Wu H., Li J., Xie J., Zang F., Maa M., Gu N., Zhang Y. (2019). Magnetic targeting combined with active targeting of dual-ligand iron oxide nanoprobes to promote the penetration depth in tumors for effective magnetic resonance imaging and hyperthermia. Acta Biomater..

[99] Gupta R., Sharma D. (2019). Biofunctionalization of magnetite nanoparticles with stevioside: Effect on the size and thermal behaviour for use in hyperthermia applications. Int. J. Hyperth..

[100] Cabada T.F., de Pablo C.S.L., Serrano A.M., del Pozo Guerrero F., Olmedo J.J.S., Gomez M.R. (2012). Induction of cell death in a glioblastoma line by hyperthermic therapy based on gold nanorods. Int. J. Nanomed..

[101] Jang Y., Lee N., Kim J.H., Park Y.I., Piao Y. (2018). Shape-Controlled Synthesis of Au Nanostructures Using EDTA Tetrasodium Salt and Their Photothermal Therapy Applications. Nanomaterials.

[102] Wang J., Zhou Z., Zhang F., Xu H., Chen W., Jiang T. (2018). A novel nanocomposite based on fluorescent turn-on gold nanostars for near-infrared photothermal therapy and self-theranostic caspase-3 imaging of glioblastoma tumor cell. Colloids Surf. B Biointerfaces.

[103] Xu H.-L., ZhuGe D.-L., Chen P.-P., Tong M.-G., Lin M.-T., Jiang X., Zheng Y.-W., Chen B., Li X.-K., Zhao Y.-Z. (2018). Silk fibroin nanoparticles dyeing indocyanine green for imaging-guided photo-thermal therapy of glioblastoma. Drug Deliv..

[104] Kang J.-H., Ko Y.T. (2019). Dual-selective photodynamic therapy with a mitochondria-targeted photosensitizer and fiber optic cannula for malignant brain tumors. Biomater. Sci..

[105] Huang J.-L., Jiang G., Song Q.-X., Gu X., Hu M., Wang X.-L., Song H.-H., Chen L.-P., Lin Y.-Y., Jiang D. (2017). Lipoprotein-biomimetic nanostructure enables efficient targeting delivery of siRNA to Ras-activated glioblastoma cells via macropinocytosis. Nat. Commun..

[106] Liu N., Zhang L., Wang Z., Cheng Y., Zhang P., Wang X., Wen W., Yang H., Liu H., Jin W. (2017). MicroRNA-101 inhibits proliferation, migration and invasion of human glioblastoma by targeting SOX9. Oncotarget.

[107] Galstyan A., Markman J.L., Shatalova E.S., Chiechi1 A., Korman A.J., Patil R., Klymyshyn D., Tourtellotte W.G., Israel1 L.L., Ljubimov BO V.A. (2019). Blood–brain barrier permeable nano immunoconjugates induce local immune responses for glioma therapy. Nat. Commun..

[108] Huang P., Wang X., Liang X., Yang J., Zhang C., Kong D., Wang W. (2019). Nano-, micro-, and macroscale drug delivery systems for cancer immunotherapy. Acta Biomater..

[109] Kadiyala P., Li D., Nuñez F.M., Altshuler D., Doherty R., Kuai R., Yu M., Kamran N., Edwards M., Moon J.J. (2019). High Density Lipoprotein-Mimicking Nanodiscs for Chemo-Immunotherapy against Glioblastoma Multiforme. ACS Nano.

[110] Abdalla AM E., Xiao L., Ullah M.W., Yu M., Ouyang C., Yang C. (2018). Current Challenges of Cancer Anti-angiogenic Therapy and the Promise of Nanotherapeutics. Theranostics.

[111] Caffo M., Cardali S.M., Fazzari E., Barresi V., Caruso G. (2018). Nanoparticles drug-delivery systems and antiangiogenic approaches in the treatment of gliomas. Glioma.

[112] Naletova I., Cucci L.M., D’Angeli F., Anfuso C.D., Magrì A., La Mendola D., Lupo G., Satriano C. (2019). A Tunable Nanoplatform of Nanogold Functionalised with Angiogenin Peptides for Anti-Angiogenic Therapy of Brain Tumours. Cancers.

[113] Sudha T., Bharali D.J., Sell S., Darwish NH E., Davis P.J., Mousa S.A. (2017). Nanoparticulate Tetrac Inhibits Growth and Vascularity of Glioblastoma Xenografts. Horm. Canc..

[114] Clavreu A., Pourbaghi-Masouleh M., Roger E., Menei P. (2019). Nanocarriers and nonviral methods for delivering antiangiogenic factors for glioblastoma therapy: The story so far. Int. J. Nanomed..

[115] Wierzbicki M., Sawosz E., Strojny B., Jaworski1 S., Grodzik M., Chwalibog A. (2018). NF-κB-related decrease of glioma angiogenic potential by graphite nanoparticles and graphene oxide nanoplatelets. Sci. Rep..

[116] Miao L., Huang L. (2015). Exploring the Tumor Microenvironment with Nanoparticles. Cancer Treat. Res..

[117] Zhu S., Gu Z., Zhao Y. (2018). Harnessing Tumor Microenvironment for Nanoparticle-Mediated Radiotherapy. Adv. Therap..

[118] Aldea M., Potara M., Soritau O., Florian I.S., Florea A., Nagy-Simon T., Pileczki V., Brie I., Maniu D., Kacso G. (2018). Chitosan-capped gold nanoparticles impair radioresistant glioblastoma stem-like cells. JBUON.

[119] Khan I.S., Ehtesham M. (2014). Targeting glioblastoma cancer stem cells: The next great hope?. Neurosurg. Focus.

[120] Lu L., Shen X., Tao B., Lin C., Li K., Luo Z., Cai K. (2019). The nanoparticle-facilitated autophagy inhibition of cancer stem cells for improved chemotherapeutic effects on glioblastomas. J. Mater. Chem. B.

[121] Singh P.K., Srivastava A.K., Dev A., Kaunda B., Choudhury S.R., Karmakar S. (2018). 1, 3β-Glucan anchored, paclitaxel loaded chitosan nanocarrier endows enhanced hemocompatibility with efficient anti-glioblastoma stem cells therapy. Carbohydr. Polym..

[122] Suryaprakash S., Lao Y.-H., Cho H.-Y., Li M., Ji H.Y., Shao D., Hu H., Quek C.H., Huang D., Mintz R.L. (2019). Engineered Mesenchymal Stem Cell/Nanomedicine Spheroid as an Active Drug Delivery Platform for Combinational Glioblastoma Therapy. Nano Lett..

[123] Elmore S. (2007). Apoptosis: A Review of Programmed Cell Death. Toxicol. Pathol..

